# L-Arginine in diabetes: clinical and preclinical evidence

**DOI:** 10.1186/s12933-023-01827-2

**Published:** 2023-04-18

**Authors:** Imma Forzano, Roberta Avvisato, Fahimeh Varzideh, Stanislovas S. Jankauskas, Angelo Cioppa, Pasquale Mone, Luigi Salemme, Urna Kansakar, Tullio Tesorio, Valentina Trimarco, Gaetano Santulli

**Affiliations:** 1 Department of Medicine, Division of Cardiology, Wilf Family Cardiovascular Research Institute, Einstein Institute for Aging Research, Fleischer Institute for Diabetes Research (FIDAM), Einstein - Mount Sinai Diabetes Research Center (ES-DRC), Albert Einstein University College of Medicine, New York, NY USA; 2 Department of Molecular Pharmacology, Institute for Neuroimmunology and Inflammation (INI), Albert Einstein College of Medicine, New York, NY, USA; 3 Montevergine Clinic, Mercogliano (AV), Italy; 4 Department of Neuroscience, Reproductive Sciences and Dentistry, “Federico II” University, Naples, Italy

**Keywords:** L-Arginine, Diabetes mellitus, Endothelial dysfunction, GLP-1, Glucose metabolism, NO

## Abstract

L-Arginine (L-Arg), is a semi-essential amino acid involved in the formation of nitric oxide. The functional relevance of L-Arg in diabetes mellitus has been evaluated both in animal models and in human subjects. In the literature there are several lines of evidence indicating that L-Arg has beneficial effects in diabetes and numerous studies advocate its administration to attenuate glucose intolerance in diabetic patients. Here we present a comprehensive overview of the main studies exploring the effects of L-Arg in diabetes, including preclinical and clinical reports on this topic.

## Introduction

L-Arginine, hereinafter referred to as L-Arg, was first isolated from lupin seeds by E. Schulze and E. Steiger in 1886, who called it “áργυρος” (argiros), a Greek word meaning silver, due to the white-silverish appearance of its crystal. L-Arg is an essential or conditionally essential amino acid — because it can be synthesized by healthy individuals but not by premature newborns [1] — that has been shown to be safe for the human body [2].

L-Arg is a natural constituent of food proteins [3]. It is elemental to produce NO, which acts as a major vasodilator with favorable effects on the cardiovascular system [4]. Moreover, L-Arg is involved in the synthesis of creatine, L-Ornithine, L-Glutamate, collagen, polyamines, and agmatine [5]. L-Arg promotes the secretion of growth hormone from the pituitary gland [6] and is implicated in T cell proliferation and host immune responses [7–9]. The intake of L-Arg has been shown to improve oxidative metabolism, through an enhanced mitochondrial function, eventually improving physical performance [10].

Several studies advocate the implementation of L-Arg for the treatment of diabetes, both directly and indirectly. L-Arg is a powerful secretagogue of the endocrine system, as it induces the secretion of insulin [11] and glucagon [12], which are protagonists in glucose metabolism. Furthermore, investigations in rats have demonstrated that L-Arg can reduce plasma glucose levels, improving glucose tolerance [13]. L-Arg supplementation was also shown to reduce adiposity and improve insulin sensitivity in animal models of obesity as well as in patients with diabetes and obesity [14]. These findings are highly relevant considering that obesity is one of the main risk factors of diabetes. Consistently, L-Arg supplementation was also found to induce a decrease in white adipose tissue (WAT) [14] and a modulation of the BAT-WAT ratio (brown adipose tissue vs. white adipose tissue) in both clinical and preclinical investigations [15–17]. Notably, Hayde and collaborators observed that oral high-dose of L-Arg supplementation has an immunomodulatory effect that could cause an enhanced clearance of advanced-stage non-enzymatic glycosylation products, thereby ameliorating glucose tolerance in diabetic patients [18]. Interesting evaluations came from molecular dynamic simulations, which revealed that it is possible to have significant effects even with the association L-Arg/metformin. Indeed, when L-Arg is combined with metformin, it is displaced from the NOS activation site, reducing nitric oxide (NO) concentration. This side effect of the association could be useful in clinical conditions in which NO could give negative consequences such as shock and stroke [19].

The main studies evaluating L-Arg supplementation in clinical trials and in animal models are reported in Tables 1 and 2, respectively.

**Table 1:** Summary of the main clinical trials investigating the effects of L-Arg supplementation in diabetes

First Author, year [Ref.]	Study design	Participants	Dose and duration of L-Arg supplementation	Main results
Lubec B, 1997 [57]	Blind placebo-controlled trial	Patients with diabetes	1 g twice/day for 3 months	No significant differences In terms of blood glucose, fructosamine, and HbA1c
Wascher T C,1997 [45].	Clinical trial	Patients with obesity and patients with T2DM	0.052 g/kg/min for 180 min	L-Arg infusion improves insulin-sensitivity in patients
Marfella R, 2000 [43]	Clinical trial	Patients with T2DM	1 g/min in infusion for 30 min	L-Arg infusion reverts the effects caused by acute hyperglycemia (increase of BP and alterations in baroreflex activity)
Piatti P.M, 2001 [46]	Double-blind trial	Patients with T2DM	3 g daily for 3 months	L-Arg administration significantly improves peripheral and hepatic insulin sensitivity in T2DM patients.
Lucotti P, 2006 [58]	Randomized controlled trial	Patients with T2DM	8.3 g daily for 21 days	L-Arg therapy improves fasting and postprandial glycemic excursions and hyperinsulinemia.
Martina V,2008 [70]	Randomized, double-blind, placebo-controlled trial	Patients with T2DM	1.2 g daily for 6 months	L-Arg improves endothelial function reducing oxidative stress and promotes NO anti-atherosclerotic effects.
Settergren M, 2008 [39]	Randomized controlled trial	Patients with T2DM and CAD	0.2 g/min in infusion for 15 min	L-Arg and BH4 administration reduces I/R-induced endothelial dysfunction
Monti L.D, 2012–2018 [53, 54]	Randomized, double-blind, placebo-controlled trials	Patients with IGT and MS	6.4 g daily for 18 months	L-Arg for 18 months significantly increases regression to NGT; 9 years from baseline the cumulative incidence of diabetes was less in the L-Arg group compared to placebo (40.6% vs. 57.4%).
Cherney D.Z.I, 2013 [62]	Clinical trial	Patients with T1DM	100 mg/kg over 30 min and then 250 mg/kg over 30 min, in infusion	L-Arg inverts the hyperglycemia renal hemodynamic effects in women
Fayh A.P, 2013 [63]	Clinical trial	Men with T1DM	7 g/day during 1 week	L-Arg improves endothelial function
Costa G, 2022 [47]	Comparative study	Women with T2DM	5 g/day for 14 days	L-Arg supplementation improves vascular and microvascular function.

BP: Blood Pressure; CAD: Coronary Artery Disease; T1DM: Type 1 Diabetes Mellitus ; T2DM: Type 2 Diabetes Mellitus; IGT: Impaired Glucose Tolerance; I/R: ischemia-reperfusion; MS: Metabolic Syndrome; NGT: Normal Glucose Tolerance.

**Table 2:** Summary of the main preclinical trials testing L-Arg effects in animal models of diabetes

First Author, year [Ref.]	Model
Alba-Roth J, 1988 [6]	Rat anterior pituitary cells co-incubated with L-Arg and GHRH for 3 h
Mohan I.K, 2000 [55]	Insulin-dependent diabetes mellitus model of alloxan-induced diabetic rats
Lass A, 2002 [56]	Rat hearts with oxygen radical-induced myocardial injury. Oxygen radicals were obtained by electrolysis or to hypoxanthine and xanthine oxidase
El Missiry M.A, 2004 [49]	Insulin-dependent diabetes mellitus model of alloxan-induced diabetic rats
Vasilijevic A, 2007 [29].	Insulin-dependent diabetes mellitus model of alloxan-induced diabetic rats
Clemmensen C, 2013 [24]	Diet-induced obese mice
Dubey H, 2022 [13]	Diet and streptozotocin induced diabetic rats

GHRH: Growth Hormone Releasing Hormone

### Role of L-Arg in glucose metabolism

**Fig. 1:**
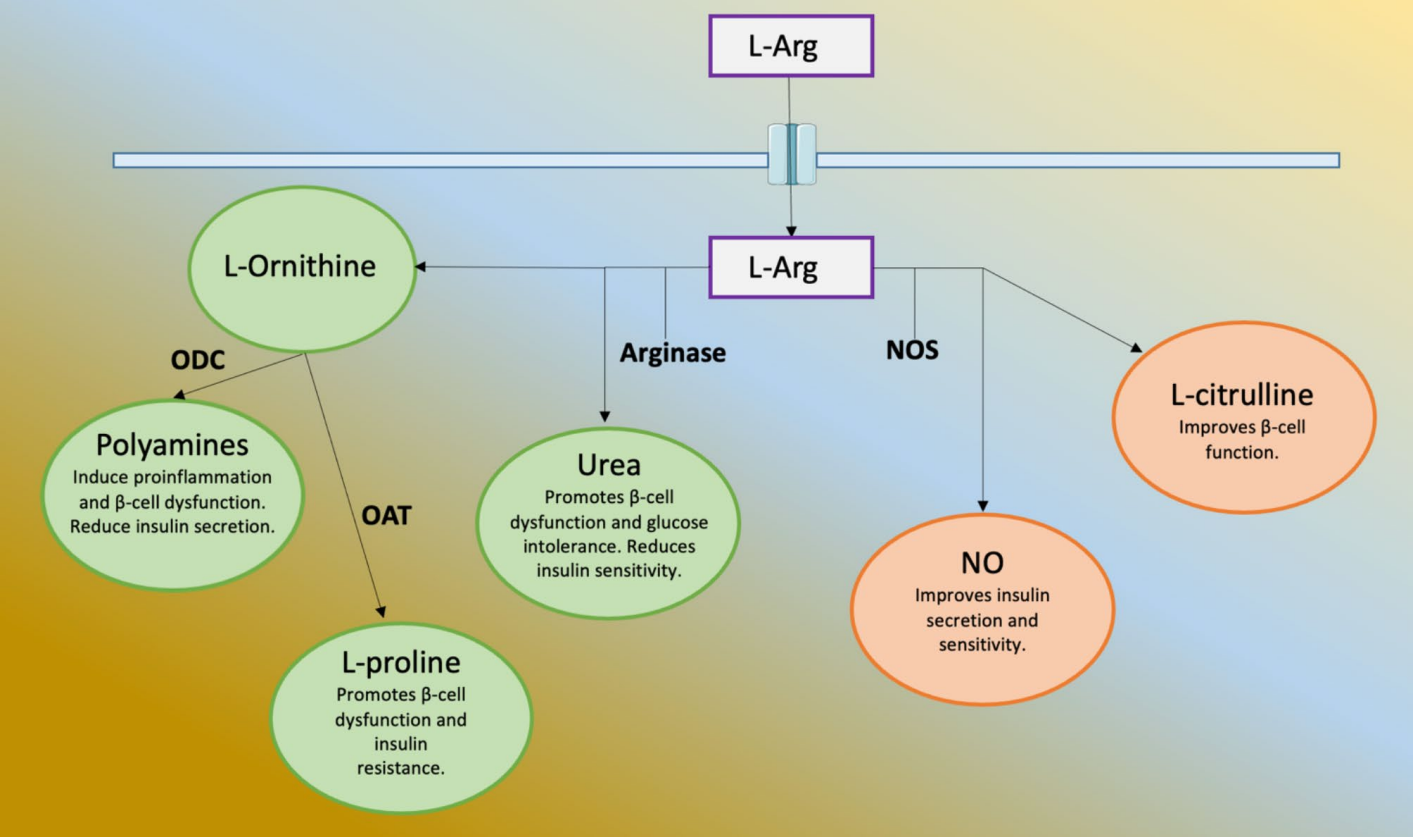
L-Arg once in the cell can be metabolized by Arginase. The products of this enzyme are: L-Ornithine, which is further cleaved to polyamines by ornithine decarboxylase (ODC), L-proline by ornithine aminotransferase (OAT), and urea. These compounds exert negative effects on glucose metabolism. When L-Arg is cleaved in nitric oxide (NO) and L-citrulline by the nitric oxide synthase (NOS), these compounds have positive effects on glucose metabolism, also exerting beneficial action on the cardiovascular system

L-Arg plays essential roles in a number of metabolic pathways, including glucose metabolism [18, 20]. For instance, L-Arg can be metabolized by both arginase and NOS. If metabolized by arginase, L-Arg is cleaved to urea and L-Ornithine, causing a dysregulation of pancreatic β-cells determining an increase of insulin resistance and glucose intolerance alongside with a pro-inflammatory state. Urea is directly involved in such effects while L-Ornithine is transformed in polyamines by ornithine decarboxylase (ODC) and in L-proline by ornithine aminotransferase (OAT). When metabolized by NOS, L-Arg produces L-citrulline and NO, with the latter being crucial for endothelial function, for insulin secretion, and improvement of insulin sensitivity [21]. Thus, in physiologic conditions, the L-Arg NOS pathway is involved in a better response to glycemic levels increasing insulin secretion and sensitivity [22, 23], as shown in Fig. 1.

### L-Arg and diabetes: preclinical evidence

In a recent study performed in rodents by Clemmensen et al. [24], L-Arg was shown to stimulate the release of Glucagon-like peptide-1 (GLP-1), an intestinal hormone that plays an important role in the regulation of appetite and glucose metabolism [25, 26]. In this work, mice harboring genetically inactivated GLP-1 receptors were compared to mice with wild-type GLP-1 receptors, implementing the feeding of both groups with L-Arg. In the first group of mice, the intake of L-Arg did not bring great improvements in glucose metabolism, while in the second group there was a marked improvement in glucose metabolism and insulin secretion. A new indirect mechanism was therefore been suggested, in which the intake of L-Arg improves glucose metabolism, insulin resistance, and insulin sensitivity [24].

Claybaugh and collaborators demonstrated, in diet-induced diabetics rats, that L-Arg supplementation can preserve NO activity. This effect could contribute to delaying the onset of insulin resistance and renal dysfunction caused by hyperglycemic stress, suggesting a main role for NO in renal function and in the pathogenesis of diabetes [27].

Similarly, L-Arg administration in diabetic rats with Alzheimer’s disease demonstrated an improvement in terms of glucose tolerance and insulin levels. Intriguingly, L-Arg exhibited ameliorative effects on cognitive deficits, suggesting a potential therapeutic action to attenuate neurological deterioration mediated by diabetes [13].

### L-Arg and diabetes: clinical trials

Drugs currently approved to treat type 2 diabetes mellitus (T2DM) mostly work by increasing insulin secretion or reducing glucose concentration but are unable to fully improve insulin sensitivity and protect beta-cells [28]. In fact, some of the main features of T2DM are the deterioration of beta-cells and the low insulin sensitivity. Conversely, L-Arg can cope with these problems since it has been shown to stimulate beta-cell neogenesis by increasing the area of beta-cells [29].

In diabetes, the condition of hyperglycemia reduces NO [10, 30], which, as mentioned above in this review, is important for the regulation of vasodilation, anticoagulation, the proliferation of smooth muscle, and the overall antioxidant capacity of endothelial cells [31–33]. L-Arg also serves as a basic substrate to produce NO in endothelial cells, thus regulating vascular tone and overall cardiovascular homeostasis [34–38]. Settergen and colleagues investigated the effect of L-Arg and tetrahydrobiopterin infusion on endothelial dysfunction induced by ischemia/reperfusion in patients with T2DM and coronary artery disease, observing that L-Arg supplementation significantly attenuated endothelial dysfunction in this type of patients [39]. Hence, an implementation of L-Arg could potentially reduce some of the main and most serious complications of diabetes, including heart failure; indeed, diabetic patients are more prone to develop cardiomyopathy than healthy subjects [40, 41].

Numerous clinical studies have confirmed the reduction of blood pressure and platelet aggregation in diabetic patients treated with intravenous L-Arg [23, 42, 43]. Additionally, the intravenous injection of L-Arg in obese T2DM patients has been shown to stimulate insulin reactivity, restoring insulin-dependent vasodilation [44, 45]. On the other hand, oral administration of L-Arg improves sensitivity to hepatic and peripheral insulin in a cGMP-dependent manner [46]. A recent comparative study has shown that oral supplementation with L-Arg (5 g/day for 14 days) improves vascular and microvascular health in elderly women with or without T2DM [47].

Other common occurrences in diabetes include oxidative stress and tissue damage [48]. In diabetic patients the redox balance is altered, leading to a high pro-oxidant enzymatic activity or a lower antioxidant enzymatic activity and is translated into augmented oxidative stress and dysfunction of endothelial cells. L-Arg has been linked to an attenuation of oxidative stress, preventing the reduction of regulation of cellular antioxidants, a finding demonstrated in a variety of species and cell lines [49–52]. Equally important, Monti and collaborators assessed the efficacy of long-term L-Arg therapy in preventing or delaying T2DM in patients with impaired glucose tolerance (IGT) and metabolic syndrome (MS); showing that the 18-month L-Arg supplementation induces a regression to normal glucose tolerance [53]. Having achieved these results, the same research grooup sought to determine whether the chronic L-Arg supplementation for 18 months maintained long-lasting effects on diabetes incidence, insulin secretion and sensitivity, oxidative stress, and endothelial function among subjects at high risk of developing T2DM. Thus, after the 18 months of L-Arg administration, people still free from diabetes were followed-up until the T2DM diagnosis. At the end of the study, the cumulative incidence of diabetes in the L-Arg group was of 40.6% and in the placebo-treated group was of 57.4%, strongly suggesting that the supplementation with L-Arg could retard the development of T2DM for a long period [54]. This effect could be linked to the L-Arg capacity of reducing oxidative stress. As discussed above, L-Arg acts as a substrate for NO, and there are in fact diverse theories regarding the L-Arg-NO system, emphasizing a protective role against oxidative stress [55, 56]. In a study authored by Lubec and co-workers, diabetic patients were treated with L-Arg for 3 months (two daily doses of 1 g), resulting in improved diabetes management, also observing a reduced lipid peroxidation [57]. Similarly, El-Missiry and collaborators reported a diminished oxidative stress following L-Arg supplementation, highlighted by lower levels of TBARS (thiobarbituric acid reactive substances), an indicator of lipid peroxidation and oxidative stress [49].

In 2006, Lucotti et al. evaluated the effects of a long-term oral L-Arg therapy. The Authors enrolled T2DM patients who followed a low-calorie diet with L-Arg (8.3 g/day) supplementation, in combination with physical training, for a period of 21 days. Such oral supplementation of L-Arg, in addition to improving endothelial function, oxidative stress, and adipokine release, ameliorated fasting glucose levels and normalized post-prandial glucose levels [58]. These results were somehow surprising, considering that previous studies had suggested that post-prandial hyperglycemia may be more significant than fasting glucose levels in terms of overall glycemic control [59–61].

In 2013, Cherney and collaborators demonstrated that L-Arg infusion reversed the exaggerated pressor response to clamped hyperglycemia in women with type 1 diabetes mellitus (T1DM), suggesting the importance of NO as a fundamental regulator of sex-dependent vascular responses to hyperglycemia [62]. In the same year, Farney and collaborators assessed in 10 men affected by non-complicated T1DM the efficacy of L-Arg administration as a critical tool for the treatment of diabetic complications, showing that L-Arg improved vascular function [63]. However, the Authors were unable to draw conclusions regarding the mechanisms by which L-Arg therapy is inducing improvements on cardiovascular function.

A recent meta-analysis of clinical trials confirmed that L-Arg is a safe compound able to reduce fasting blood glucose and serum insulin levels in patients with alterations of glucose metabolism [64].

### Potential issues associated with oxidative stress

A problem resulting from the intake of L-Arg could be the risk of reaction with precursors of advanced glycosylated products [65] that are found to be abundant in diabetic patients. It has been shown in vivo that reacting with methylglyoxal, a molecule abundant in diabetic patients [66, 67], L-Arg might produce powerful superoxide radicals [68]. For this reason, it has been suggested to combine an antioxidant with the implementation of L-Arg [69, 70]. This aspect was confirmed in a study where 24 patients received oral treatment of L-Arg combined with N-acetylcysteine; such a treatment led to a reduction of blood pressure, total cholesterol, reactive C-proteins, and vascular adhesion molecules, all well-established risk factors for diabetes [70].

## Conclusions

Overall, data currently available in the literature consider L-Arg supplementation safe and significant for the treatment of diabetes. Based on these considerations, L-Arg could represent an additional strategy for patients with diabetes, especially in the early stages of the disease, in order to prevent or at least slow down complications involving other organs. Among the various L-Arg formulations currently available, using the ones with a standardized formula in oral vials with no sugar should be preferred. Further randomized and long-term placebo-controlled clinical trials are warranted to definitively assess L-Arg beneficial effects on glucose metabolism and to define the cellular and molecular mechanisms underlying the metabolic benefits of L-Arg in diabetes.

## Data Availability

N/A.
