# Correction: L-Arginine in diabetes: clinical and preclinical evidence

**DOI:** 10.1186/s12933-023-01852-1

**Published:** 2023-05-18

**Authors:** Imma Forzano, Roberta Avvisato, Fahimeh Varzideh, Stanislovas S. Jankauskas, Angelo Cioppa, Pasquale Mone, Luigi Salemme, Urna Kansakar, Tullio Tesorio, Valentina Trimarco, Gaetano Santulli

**Affiliations:** 1 Department of Medicine, Division of Cardiology, Wilf Family Cardiovascular Research Institute, Einstein Institute for Aging Research, Fleischer Institute for Diabetes Research (FIDAM), Einstein - Mount Sinai Diabetes Research Center (ES-DRC), Albert Einstein University College of Medicine, New York, USA; 2 Department of Molecular Pharmacology, Institute for Neuroimmunology and Inflammation (INI), Albert Einstein University College of Medicine, New York, USA; 3 Montevergine Clinic, Mercogliano, (AV), Italy; 4 Department of Neuroscience, Reproductive Sciences and Dentistry, “Federico II” University, Naples, Italy

**Correction: Cardiovascular Diabetology (2023) 22:89**

10.1186/s12933-023-01827-2

Following publication of the original article [1], the conflict of interest statement has been updated with this correction. 

**Conflict of Interest**

Gaetano Santulli declares that he is Associate Editor of Cardiovascular Diabetology and that the article was assigned to another Editor to assume responsibility for overseeing peer review. This submissions was subject to the exact same review process as any other manuscript submitted to the journal.
